# Obesity Impairs Skin Barrier Function and Facilitates Allergic Sensitization in Mice

**DOI:** 10.1111/all.70067

**Published:** 2025-09-23

**Authors:** Alicia Martinek, Andrea Deinzer, Roman G. Gerlach, Jana Petzold, Lea Semmler, Christof Vorsatz, Padraic G. Fallon, Christian Schwartz

**Affiliations:** ^1^ Mikrobiologisches Institut—Klinische Mikrobiologie, Immunologie und Hygiene Universitätsklinikum Erlangen and Friedrich‐Alexander‐Universität (FAU) Erlangen‐Nürnberg Erlangen Germany; ^2^ FAU Immunomedicine (FAU I‐MED) Erlangen Germany; ^3^ Trinity Biomedical Sciences Institute, School of Medicine Trinity College Dublin Dublin 2 Ireland

**Keywords:** atopic dermatitis, obesity, skin barrier

## Abstract

Atopic dermatitis is a chronic inflammatory skin condition marked by intense itching and a weakened skin barrier. The compromised skin barrier often leads to exaggerated immune responses and greater sensitivity to allergens. Previous studies have already implicated a link between obesity and atopic dermatitis; however, the mechanisms linking obesity to atopy are not yet well understood. We propose that obesity impairs skin barrier function, facilitating allergen penetration in the skin and triggering systemic and local allergic sensitization. We used a diet‐induced obesity mouse model to examine skin barrier integrity and immune responses in both steady‐state and inflammatory conditions. In order to induce dermatitis or food allergy, we epicutaneously applied MC903 or ovalbumin, respectively. We observed that obesity significantly alters skin barrier physiology, as indicated by increased transepidermal water loss in obese animals. Over time, we observed a decrease in key skin barrier proteins—preceding overt cutaneous inflammation, further indicating a loss of barrier integrity during obesity. Interestingly, skin barrier breakdown was independent of changes to the microbiome. On a cellular level, immune profiling revealed a shift towards a type 17 helper T‐cell response bias, although this shift did not coincide with an increase in cytokine production under steady‐state conditions. Topical application of MC903 in obese animals led to increased ear swelling and a pronounced Th17‐biased inflammatory response compared to lean counterparts. Our findings show that obesity weakens the skin barrier, facilitating increased allergen penetration and allergic sensitization. The Th17‐skewed immune environment in obese animals may also amplify inflammatory responses to allergens and act as a feed‐forward loop to further disintegrate the skin barrier. This study highlights how obesity‐induced skin barrier dysfunction contributes to allergic conditions like atopic dermatitis and may be therapeutically targeted by barrier restoration.

## Graphical abstract

Diet‐induced obesity increases transepidermal water loss and compromises barrier function through downregulation of tight junctions and desmosomes. During obesity, MC903 elicits a mixed Th1/2/17 immune response in the skin leading to aggravated ear swelling. The compromised skin barrier during obesity facilitates allergen penetration and cutaneous sensitization. Abbreviations: MC903, a synthetic vitamin D3 analogue; OVA, ovalbumin; TEWL, transepidermal water loss; Th, T helper cell.

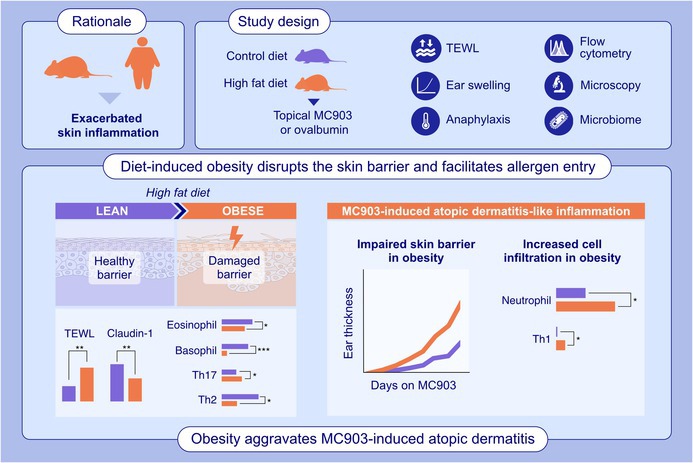

## 1 Introduction

Obesity has become a significant public health concern, with its rising prevalence linked to various comorbidities, including atopic dermatitis (AD). AD is a chronic inflammatory skin condition characterized by pruritus and eczematous lesions, often exacerbated by environmental factors and immune dysregulation [1]. The relationship between obesity and AD is complex, with evidence suggesting that obesity may increase the risk of developing AD and worsen its severity [2, 3]. This association is particularly concerning given the globally increasing rates of obesity, which may contribute to the rising incidence of allergic diseases. Atopic dermatitis is primarily driven by a dysfunctional skin barrier, which allows allergens and irritants to penetrate the skin, triggering inflammatory responses. A compromised skin barrier is characterized by increased transepidermal water loss (TEWL), leading to dryness and irritation [4, 5]. This barrier dysfunction is often associated with the activation of various immune cells, including type 2 T helper cells (Th2), Th17 cells, eosinophils, basophils, and type 2 innate lymphoid cells (ILC2s). These cells play a crucial role in the pathogenesis of AD by producing pro‐inflammatory cytokines such as interleukin (IL)‐4, IL‐5, and IL‐13, which further exacerbate skin inflammation and contribute to barrier impairment [6, 7]. The interplay between obesity, skin barrier integrity, and allergic sensitization highlights the need for further investigation into the mechanisms underlying these associations.

Obesity is associated with systemic inflammation and dysbiosis, which may compromise skin barrier function [8]. Adipose tissue in obese individuals produces pro‐inflammatory mediators, such as leptin and TNF‐α, that exacerbate inflammation not only locally but also systemically [9]. This inflammatory milieu can lead to dysfunction in skin immune cells, such as dendritic cells [10, 11] or γδ T cells, which are crucial for maintaining keratinocyte homeostasis [12]. In obese conditions, these γδ T cells exhibit impaired functionality, contributing to skin barrier dysfunction and increased susceptibility to inflammatory skin diseases like psoriasis and eczema [12]. Similarly, T cells in the skin are more prone to promote AD‐like inflammation in a mouse model via a mixed Th1/Th17 response instead of the “classical” Th2 response towards MC903 treatment [13].

We therefore hypothesize that obesity impairs skin barrier integrity, which leads to facilitated allergic sensitization in part mediated by an altered cutaneous immune response. To address this, we aimed to (1) characterize structural and functional changes in the skin barrier of mice subjected to a high‐fat diet (HFD) compared to animals on a control diet (CD); (2) assess the immune response of obese mice in a model of MC903‐induced AD‐like inflammation; and (3) evaluate the functional consequences of skin barrier disruption using a model of epicutaneous sensitization with ovalbumin (OVA). In addition, we investigated whether the observed effects were dependent on the skin microbiome. Our approach allowed us to dissect the contribution of obesity to skin barrier dysfunction and its role in cutaneous allergic sensitization.

## 2 Methods

### 2.1 Study Design

The study employed a diet‐induced obesity mouse model, initiating with male C57BL/6J mice aged 6–8 weeks, which were then maintained on either a HFD or CD for periods spanning 2–14 weeks. Throughout the study, various measurements were taken at different intervals: body weight was recorded weekly; TEWL was assessed biweekly to monitor skin barrier function. For gene expression analysis, mice were analyzed before and after 2, 6, 10, and 14 weeks on HFD. To induce allergic responses, mice were treated after 8 weeks on HFD or CD and maintained on the respective diet. Ear thickness was measured daily for 7 days during the MC903‐induced dermatitis phase. Flow cytometry was conducted on ear and ear‐draining lymph node samples at the conclusion of the MC903 treatment (day 7) and following ovalbumin challenge (day 17); ELISA for cytokine detection was performed on restimulated lymph node cells after 72 h, and OVA‐specific antibodies were measured in serum samples 3 days post‐final OVA challenge.

### 2.2 Mice

Male C57BL/6J mice were purchased from Charles River (Sulzfeld, Germany). Animals were housed in a specific pathogen–free facility. Mice had ad libitum access to food and water, and welfare was checked daily. All animal experiments were performed in compliance with German animal protection law and approved by the Federal Government of Lower Franconia (RUF‐55.2.2‐2532‐2‐1177 and –1813).

### 2.3 Diet‐Induced Obesity

Age‐matched mice starting at 6–8 weeks of age were kept on a high‐fat diet (60% kcal fat, D12492; ssniff Spezialdiäten, Soest, Germany) or a control diet (10% kcal fat) for 2–14 weeks as indicated. Body weight was recorded weekly. In order to assess the impact of the microbiome on diet‐induced obesity, broad‐spectrum antibiotics (ampicillin, gentamicin, metronidazole, neomycin, vancomycin) were administered twice weekly by oral gavage.

### 2.4 Transepidermal Water Loss Measurement

TEWL was measured using a TEWAmeter (Courage+Khazaka electronic GmbH, Cologne, Germany) to assess skin barrier function by quantifying water evaporation from the skin surface in g/h/m^2^. The nano‐probe was placed for at least 60 s on the dorsal side of the ear of isoflurane‐anesthetized animals to allow for stabilization of the readings. TEWL was recorded using the MPA CT Plus software.

### 2.5 Topical Application of MC903

MC903, a synthetic vitamin D3 analogue, has been effectively utilized in mouse models to induce AD‐like skin lesions, characterized by increased ear thickness, erythema, xerosis, and significant scratching behaviours [7, 14, 15]. These lesions are associated with heightened type 2 cytokine responses and the activation of innate lymphoid cells, reflecting the immunological and histopathological features comparable to human AD [7, 16]. Following 8 weeks of HFD—a time point when the skin barrier was already impaired—mice received daily topical applications of 4 nmol MC903 (Cayman Chemical, Ann Arbor, MI) in ethanol onto the dorsal side of the ears for 7 days. Under light anesthesia with isoflurane, ear thickness was measured daily, using a digital thickness gauge (INSIZE, Siegen, Germany). On day 7, ears and ear‐draining lymph nodes were dissected and prepared for histology, RNA, or flow cytometry.

### 2.6 Epicutaneous Sensitization With Ovalbumin

Mice were kept on CD or HFD for 12 weeks. Then ovalbumin (10 mg/mL; Sigma) in 10 μL oil was applied to the dorsal side of the ears on Days 0, 3, 5, 8, 10, and 12. As a positive control, we tape‐stripped mice on CD to break the skin barrier. Therefore, mice were anesthetized with isoflurane, and adhesive tape was used to tape‐strip the dorsal ear skin for six times every other day for 2 weeks before OVA was applied epicutaneously to the ear. Blood was drawn from the *Vena facialis* 3 days after the final OVA challenge to measure OVA‐specific IgG1 and IgE in the serum. Four days after the final OVA challenge, mice received 100 mg OVA in 200 μL PBS by oral gavage. Rectal temperature was measured before and every 10 min after the OVA challenge.

### 2.7 Sample Preparation

Mice were killed by cervical dislocation. Ears were cut off at the base above the fur line, and skin (4 × 4 cm^2^) was obtained from the flank after removing hair with depilatory cream. Auricular and inguinal lymph nodes were collected. Ears were separated into dorsal and ventral halves. Skin and ear were then minced into fine pieces in HBSS medium to facilitate tissue digestion. After mincing with scissors, skin and ear samples were incubated in a thermal shaker at 37°C for 90 min with collagenase D (0.5 mg/mL), DNase I (0.2 μg/mL), and liberase (0.25 mg/mL) in HBSS. After digestion, tissue was filtered through a 100 μm cell strainer using a syringe plunger. Lymph nodes were filtered through a 70 μm strainer using the back of a syringe.

### 2.8 Flow Cytometry

Single cell suspensions were counted and stained with Zombie aqua fixable dye (Biolegend) in PBS for 30 min at 4°C, and Fc receptors were blocked with 10 μg/mL purified anti‐CD16/32 antibody (clone 93; eBioscience) in FACS buffer (PBS, 2% FBS, and Na_3_N) for 5 min at RT. After washing with FACS buffer, cells were stained for 20 min at 4°C with antibodies listed in Table 1.

**TABLE 1:** Antibodies.

Antigen	Antibody	Clone	Source
CD11b	BUV737	M1/70	BD Biosciences
CD11c	APC	N418	Biolegend
CD200R3	PE	Ba13	Biolegend
CD206	PE‐Cy7	C068C2	Biolegend
CD4	APC fire750	RM4‐5	Biolegend
CD44	BUV737	IM7	BD Biosciences
CD45	PerCP Cy5.5	30‐F11	Biolegend
CD45	BUV605	30‐F11	Biolegend
CD49b	Alexa 647	Hmα2	Biolegend
CD62L	BV785	MEL‐14	Biolegend
CD64	BV711	X54‐5/7.1	Biolegend
CD8	PerCPCy5.5	53–6.7	Biolegend
CD90.2/Thy1.2	FITC	53–2.1	Biolegend
F4/80	PE‐Dazzle 594	BM8	Biolegend
FoxP3	PE‐e610	FJK‐16s	Invitrogen
GATA3	PE	TWAJ	Invitrogen
Live‐dead	Zombie aqua	—	Biolegend
Ly6C	BV605	HK1.4	Biolegend
Ly6G	APC‐e780	1A8	BD Biosciences
MHC2	BV785	M5/114.15.2	Biolegend
PD‐L2	FITC	122	Invitrogen
RORγt	BV421	Q31‐378	BD Biosciences
Siglec‐F	BV421	E50‐2440	BD Biosciences
ST2	PE‐Cy7	DIH9	Biolegend
Tbet	APC	4B10	Biolegend

Basophils were defined as CD45^+^CD49b^+^CD200R3^+^, eosinophils as CD45^+^Siglec‐F^+^CD11b^+^SSC^hi^, neutrophils as CD45^+^Ly6G^+^, classically activated macrophages (CAM) as CD45^+^CD64^+^Ly6C^hi^ and alternatively activated macrophages (AAM) as CD45^+^CD64^+^CD206^+^/PD‐L2^+^. For intracellular staining of transcription factors, we used the FoxP3 staining buffer kit (eBioscience) according to the manufacturer's instructions. Th1 cells were identified as CD45^+^CD4^+^Tbet^+^, Th2 cells as CD45^+^CD4^+^GATA3^+^, Th17 cells as CD45^+^CD4^+^Rorγt^+^, regulatory T cell (Treg) as CD45^+^CD4^+^Foxp3^+^, ILC2 as CD45^+^lin^−^Thy1^+^GATA3^+^.

Samples were acquired on a Northern Lights spectral flow cytometer (Cytek Biosciences, Fremont, CA). Fluorescence Minus One controls (FMO) were used to ensure correct gate setting. Data were analyzed by FlowJo software (version 10.10).

### 2.9 ELISA

Lymph nodes (2 × 10^5^ cells) were restimulated with 1 μg/mL coated anti‐CD3 (clone: 145‐2C11) and 0.5 μg/mL anti‐CD28 in RPMI 1640 (+10% FCS, L‐glutamine, penicillin/streptomycin) supplemented with 20 ng/mL IL‐2. Cells were incubated at 37°C, 5% CO_2_ for 72 h. The IL‐17A and IL‐13 ELISAs (Peprotech) were performed following the manufacturer's instructions.

In order to detect OVA‐specific antibodies, plates were coated with 50 μL chicken OVA (Sigma) in PBS (20 μg/mL) and incubated overnight in a humid chamber at 4°C. Plates were washed with washing buffer (0.05% Tween‐20 in PBS) and subsequently blocked with 3% BSA in PBS for 2 h. After washing, serum samples were applied and incubated for 2 h at RT. Alkaline phosphatase‐conjugated anti‐IgG1 (Southern Biotech) in 1% BSA in PBS was added to the samples and incubated for 1 h. pNPP was used as substrate, and absorbance was measured at 405 nm.

### 2.10 Rt‐PCR

Organs used for RNA isolation were snap frozen in liquid nitrogen and stored at −80°C until RNA isolation. For RNA isolation, we used Trizol and stainless‐steel beads (5 mm) in a screw cap microfuge tube. The tissue was ruptured using the Bead Ruptor 24 (Omni International, Kennesaw, GA). Samples were transferred into 1.5 mL Eppendorf tubes, and RNA was isolated using the chloroform method. The concentration of RNA was measured on an ND‐100 spectrophotometer (Nanodrop). RNA was reverse transcribed into cDNA using the high‐capacity cDNA reverse transcription kit (Applied Biosystems). RT–PCR was conducted using the SYBR select master mix (Life Technologies) on a ViiA7 Real‐Time PCR system. Primers for target (*Il13, Il33, Il17a, Il17f, Flg, Cldn1, Lep, Adipoq, Dsg3, Dsg1, Ivl, S100a9, Ocln*) and housekeeping genes (*Hprt1*) are listed in Table 2.

**TABLE 2:** Primer sequences.

Primer	Sequence
*Hprt1 fwd*	TCAGTCAACGGGGGACATAAA
*Hprt1 rev*	GGGGCTGTACTGCTTAACCAG
*Il13 fwd*	CCTGGCTCTTGCTTGCCTT
*Il13 rev*	GGTCTTGTGTGATGTTGCTCA
*Il33 fwd*	TCCAACTCCAAGATTTCCCCG
*Il33 rev*	CATGCAGTAGACATGGCAGAA
*Il17a fwd*	CACTTTGCCTCCCAGATCAC
*Il17a rev*	ACCAATCCCAAAAGGTCCTC
*Il17f fwd*	CTCTGTGTGAAGGCCGATCTC
*Il17f rev*	TGCCATGCACACCTTACTGAG
*Filaggrin fwd*	ATGTCCGCTCTCCTGGAAAG
*Filaggrin rev*	TGGATTCTTCAAGACTGCCTGTA
*Claudin‐1 fwd*	GGGGACAACATCGTGACCG
*Claudin‐1 rev*	AGGAGTCGAAGACTTTGCACT
*Leptin fwd*	GAGACCCCTGTGTCGGTTC
*Leptin rev*	CTGCGTGTGTGAAATGTCATTG
*Adiponectin fwd*	TCTTCGGGATGTTCTTCCTGG
*Adiponectin rev*	TTTGGAAAAAGTCCGAGAGACC
*Desmoglein‐1 fwd*	ACTGTGTTAAATGTCATCGAGGG
*Desmoglein‐1 rev*	TGCCTGTTCTTGAGTCAACAAC
*Desmoglein‐3 fwd*	TGGCAGTCTGGAAGTCACC
*Desmoglein‐3 rev*	CTGTAGAGGGTCAGGGATGG
*Involucrin fwd*	ATGTCCCATCAACACACACTG
*Involucrin rev*	TGGAGTTGGTTGCTTTGCTTG
*S100a9 fwd*	CTCTAGGAAGGAAGGACACC
*S100a9 rev*	GCCATCAGCATCATACACTC
*Occludin fwd*	CAGGTGAGCACCTTGGGATTCC
*Occludin rev*	CTCACGGACATGGCTGATGTCAC

### 2.11 Skin Explant and Keratinocyte Culture

Round skin punch biopsies (approx. 1 cm^2^ obtained from the flank) were cultured on gelatine sponges (Cutanplast, Henry Schein Dental, Germany) in 24‐well plates at 37°C for 3–5 days. The dermal side of the skin biopsy was placed on the surgical sponge, which was soaked with EpiLife medium (Gibco). The explant was maintained on the air‐liquid interface, not touching the medium directly. Medium was changed every 2–3 days, and sponges were replaced every third day.

Keratinocytes were obtained from the tails of adult mice as previously described [17]. In brief, tail skin of adult mice was digested with 4 mg/mL dispase (Gibco) in KC basal medium (Thermo) overnight at 4°C. Epidermis was removed from the dermis and treated with TrypLE (Thermo) for 20 min on a horizontal shaker. Epidermal cells were then physically released from the skin and cultured in keratinocyte growth medium (KC basal medium, HKGS, penicillin/streptomycin, 250 μg/mL amphotericin B) on 24 well plates pre‐coated with collagen (5 μg/cm^2^) at a density of 10 × 10^4^ cells/cm^2^ for 5 days. Medium was renewed on Day 1 and Day 3. On Day 5, cells were terminally differentiated into keratinocytes by adjusting CaCl_2_ to 0.2 nM for 48 h.

### 2.12 Histology and Immunofluorescence Microscopy

Ears and skin were embedded in Tissue Tek after fixation in 4% PFA. Skin was placed on a small piece of cardboard before fixation in order to maintain structure. Tissues were sectioned using a CryoStar NX70 cryostat. 7–9 μm thick slices were collected on glass slides for further routine hematoxylin and eosin (H&E) staining. For immunofluorescence staining, tissue sections were blocked with rat serum and 1% BSA before staining with rabbit‐anti‐ZO‐1 (Abcam) and mouse‐anti‐Claudin‐1 antibody (Invitrogen), followed by secondary antibodies AlexaFlour488‐conjugated donkey‐anti‐rabbit IgG (Jackson) and AlexaFlour647‐conjugated donkey‐anti‐mouse IgG (Jackson). Stained sections were then mounted with DAPI‐containing Fluoroshield. Images were acquired on a Keyence BZ‐X810. Images were analyzed using ImageJ (1.54p; Fiji) software by analyzing at least three stained sections per mouse from four mice per group and two independent experiments.

### 2.13 16S rRNA Gene Sequence Analysis

For analysis of the microbial composition, DNA was isolated from skin swabs and fecal pellets of WT mice on HFD or CD using the Quick‐DNA Fecal/Soil Microbe Miniprep Kit or the Quick‐DNA/RNA Miniprep Plus Kit (Zymo Research) following the manufacturer's instructions. Amplification of the 16S rRNA gene, library preparation, and sequencing were done as described elsewhere [18], but using primers 27Fmod 5′ AGRGTTTGATCMTGGCTCAG 3′ and 519R 5′ GWATTACCGCGGCKGCTG 3′ targeting the 16S V3–V4 region. Zero‐radius operational taxonomic units (zOTUs) were determined using USEARCH v11 [19] through the IMNGS server [20]. Taxonomic classification of the identified zOTUs was done using SINA [21] with SILVA database release 138.2 and NCBI nucleotide BLAST [22]. All further analyses were performed with custom scripts utilizing the phyloseq package [23] within R v4.5.1 [24]. Sequencing data are deposited under BioProject accession number PRJNA1299357.

### 2.14 Statistical Analysis

Mice were randomly assigned to treatment and control groups. Flow cytometry data were analyzed using FlowJo software (Treestar, v10.10). GraphPad Prism Software (v10) was used to perform statistical analysis. Outliers in our data sets were identified by the Rout method, and normal distribution was determined by the Shapiro–Wilk test. Mean and SEM were calculated from repeated experiments. To determine statistical significance, one‐way ANOVA with Tukey's multiple comparison test, Student's *t*‐test, or Mann–Whitney *U*‐test was performed. Statistical significance was defined as *p* < 0.05. Only relevant statistics are displayed in the figures.

## 3 Results

### 3.1 Obesity Disrupts Skin Barrier Integrity

**FIGURE 1:**
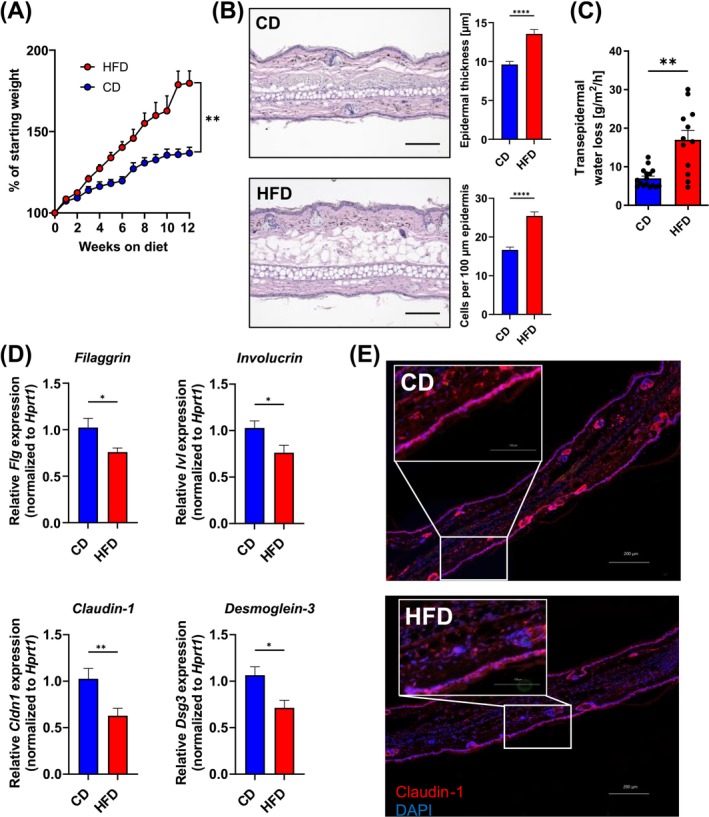
High‐fat diet impairs the skin barrier. (A) Weight gain of mice on high‐fat diet (HFD, red; *n* = 17) or control diet (CD, blue; *n* = 10) as percent increase of the starting weight (=100%). Graph shows the mean + SEM. (B) Representative hematoxylin and eosin (H&E) stained paraffin sections of ear skin of mice kept on CD (top) or HFD (bottom) for 12 weeks. Scale bar represents 100 μm. Bar graphs show the epidermal thickness (top) and cells per 100 μm epidermal segment in mice on CD (blue) or HFD (red). Data show the mean + SEM of four mice (three segments per mouse) per group from two independent experiments. (C) Transepidermal water loss (TEWL) measurement of mice kept on CD (blue) or HFD (red) for 12 weeks. Bars show the mean + SEM of 12–17 mice per group from three independent experiments. (D) Expression of indicated genes in the ear skin of mice kept on CD (blue) or HFD (red) for 12 weeks. Bars show the mean + SEM of 7 mice per group from two independent experiments. (E) Immunofluorescence staining of cryo‐sections of ears from mice kept on CD or HFD. Red = Claudin‐1, blue = DAPI. Bar = 200 μm, *p < 0.05, **p < 0.01, Student's *t*‐test (A: Of area under curve; B–D).

Obesity is associated with the development of skin inflammation, such as psoriasis and atopic dermatitis. Here, the skin barrier is often disrupted and poses a risk factor for the development of allergy. In order to address the question of whether obesity has an impact on the skin barrier integrity in mice, we kept mice on a high‐fat diet for 12 weeks (Figure 1A). The H&E staining of skin from mice on HFD revealed, in addition to the anticipated expansion of the adipose tissue layer, notable alterations in the epidermis that were characterized by increased thickness and marked cellular infiltration (Figure 1B). Therefore, we measured skin barrier integrity biweekly using transepidermal water loss (TEWL) as an indicator of a leaky skin barrier. Indeed, TEWL was significantly (*p* = 0.0018) increased from 7 g/m^2^/h on CD to 17 g/m^2^/h from 12 weeks after starting the obesogenic diet (Figure 1C). Furthermore, we evaluated the expression of genes associated with an intact skin barrier function. Filaggrin is a critical protein in the maintenance of an intact skin barrier. Here, we observed a significant reduction of *Flg*, indicating a loss of skin integrity (Figure 1D). Similarly, transcripts of involucrin, which provides structural support to corneocytes, claudin‐1, a component of tight junctions, and desmoglein‐3, a transmembrane glycoprotein component of desmosomes, were all significantly down‐regulated in mice kept on HFD (Figure 1D), while we did not observe altered *Dsg1* expression (Figure S1). Using immunofluorescence staining of claudin‐1 and the tight junction protein Zonula occludens‐1 (ZO‐1), we observed decreased expression in the skin of mice on HFD compared to CD (Figure 1E and Figure S2). These results indicate that HFD feeding leads to a significant impairment of skin barrier function.

### 3.2 Skin Barrier Proteins Are Downregulated Early During Diet‐Induced Obesity

**FIGURE 2:**
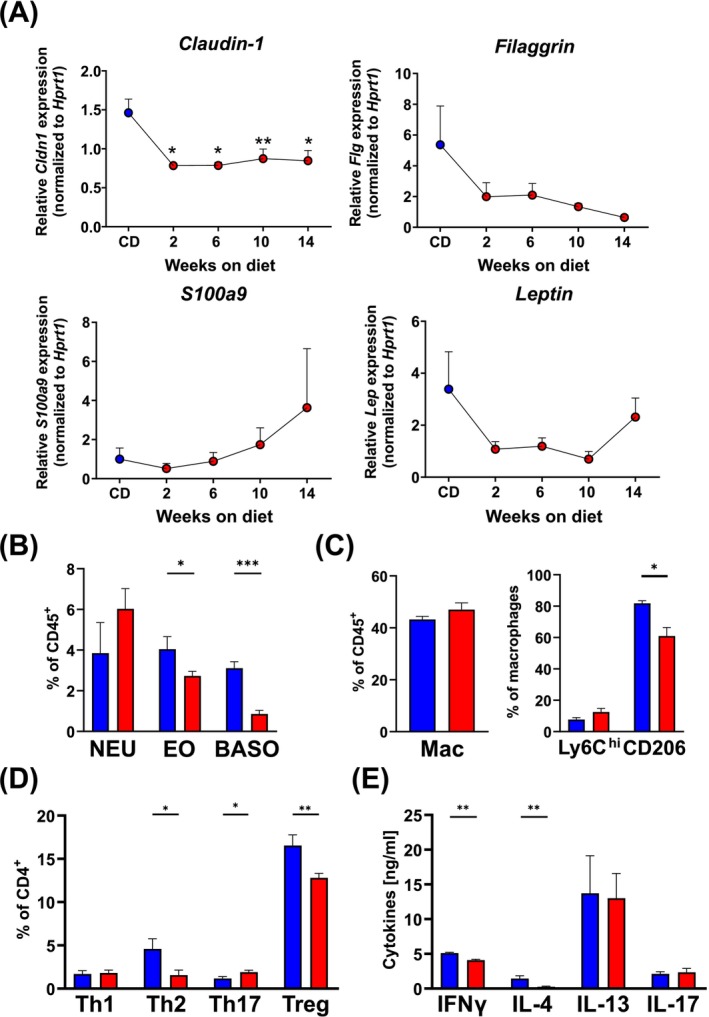
High‐fat diet promotes early skin barrier breakdown and immune dysregulation. (A) Expression of indicated genes in the ear skin of mice kept on CD (blue) or HFD (red) at the indicated time points. Depicted is the mean + SEM of 6–12 mice per group from two independent experiments. Significance was determined against mice on CD: *p < 0.05, **p < 0.01; One‐way ANOVA. (B–D) Flow cytometric analysis of neutrophils (NEU), eosinophils (EO) and basophils (BASO) (B), macrophages (Mac) as well as their expression of Ly6C and CD206 (C), and T helper cell subsets (D) in the ear skin of mice kept on CD (blue) or HFD (red) for 12 weeks. (E) IFNγ‐, IL‐4‐, IL‐13‐ and IL‐17A‐production of restimulated (anti‐CD3/anti‐CD28; 72 h) skin‐draining lymph nodes determined by ELISA. (B–E) Bars show the mean + SEM of 6–8 mice per group from two independent experiments. *p < 0.05, **p < 0.01, ***p < 0.001, Student's *t*‐test.

We were interested to explore how fast the skin barrier becomes disrupted when mice become obese. Measurement over time revealed that TEWL was already increased at 2 weeks after the start of HFD, with significant increases from 4 weeks (Figure S3). These changes in TEWL in HFD‐treated mice were reflected in the rapid downregulation of skin barrier gene expression (Figure 2A). Tight junctions are impaired as early as 2 weeks on HFD, with the expression of filaggrin in the skin also declining after 2 weeks. Interestingly, with increased time on HFD, the antimicrobial peptide S100a9 gets upregulated, indicating an ongoing antibacterial response—probably induced by a shift in the skin microbiome, with bacteria now being able to infiltrate into deeper layers of the compromised skin during obesity. Leptin expression only starts to increase after 14 weeks on HFD, which may indicate a limited role in early immunomodulation of the skin.

We further investigated whether the defective skin barrier in obesity affects immune cell populations in the skin and skin‐draining lymph nodes (Figure 2B–F). Indeed, we could observe a shift towards pro‐inflammatory neutrophils (Figure 2B) and Ly6C^hi^ macrophages (Figure 2C), while type 2‐associated cells such as eosinophils and basophils (Figure 2B), as well as anti‐inflammatory alternatively activated macrophages (AAMs) (Figure 2C), are decreased. This pro‐inflammatory shift is also reflected in the decreased frequency of Th2 cells (4.6% on CD vs. 1.6% on HFD; *p* < 0.05) and Tregs (16.5% on CD vs. 12.8% on HFD; *p* < 0.05), while Th17 cells increase in the skin of HFD‐fed mice (1.1% on CD vs. 1.9% on HFD; *p* < 0.05) (Figure 2D). However, without further stimuli, T cells in the draining lymph nodes do not differ in the production of IL‐13 and IL‐17A cytokines, while IL‐4 and IFNγ production decreased during HFD (Figure 2E).

### 3.3 Obesity Changes the Skin Microbiome

**FIGURE 3:**
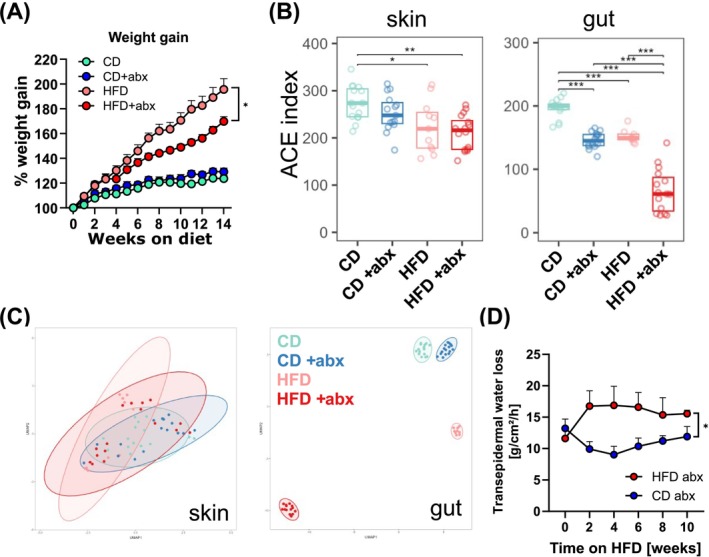
Obesity impairs the skin barrier independent of the microbiome. (A) Weight gain of mice kept on CD (green) or HFD (light red) alone or were treated with antibiotics (CD+abx: Blue; HFD+abx: Red). Graph shows the mean + SEM of 6 mice per group from two experiments. **p < 0.01; ANOVA of area under the curve; only relevant statistics are shown. (B) Analysis of alpha‐diversity measured by Abundance‐based coverage estimator (ACE) index of 16S rRNA analysis of skin swabs and fecal pellets from mice shown in (A) after 8 weeks on respective treatments. (C) Beta‐diversity of skin and gut microbiome depicted as UMAP of generalized UniFrac distance of CLR‐transformed ZOTUs/ASVs count data of groups shown in (A). (D) TEWL of mice shown in (A) was measured at indicated time points after start of the respective diets/treatment. Graph shows the mean + SEM of 6 mice per group from two experiments. *p < 0.05; ANOVA of area under the curve.

In order to determine the impact of the microbiome on the obesity‐induced skin barrier impairment, mice on their respective diets were treated with a broad‐spectrum antibiotic cocktail. Interestingly, mice on HFD that were treated with oral antibiotics had a slower weight gain than mice on HFD (Figure 3A). The efficacy of the oral antibiotic regimen was confirmed, as antibiotics successfully reduced the intestinal microbiome in mice on both CD and HFD (Figure 3B). Skin microbial composition also changed in response to oral antibiotic treatment, albeit to a lesser extent compared to changes in the gut. Interestingly, antibiotic treatment affected mice markedly more on HFD than mice on CD, which is also reflected in the analysis of beta diversity (Figure 3C). Importantly, diet had a stronger impact on microbial composition than antibiotic treatment. However, the skin barrier breakdown in obese mice on a HFD occurred independently of changes to the microbiome (Figure 3D).

### 3.4 Obesity Facilitates Allergen Entry

**FIGURE 4:**
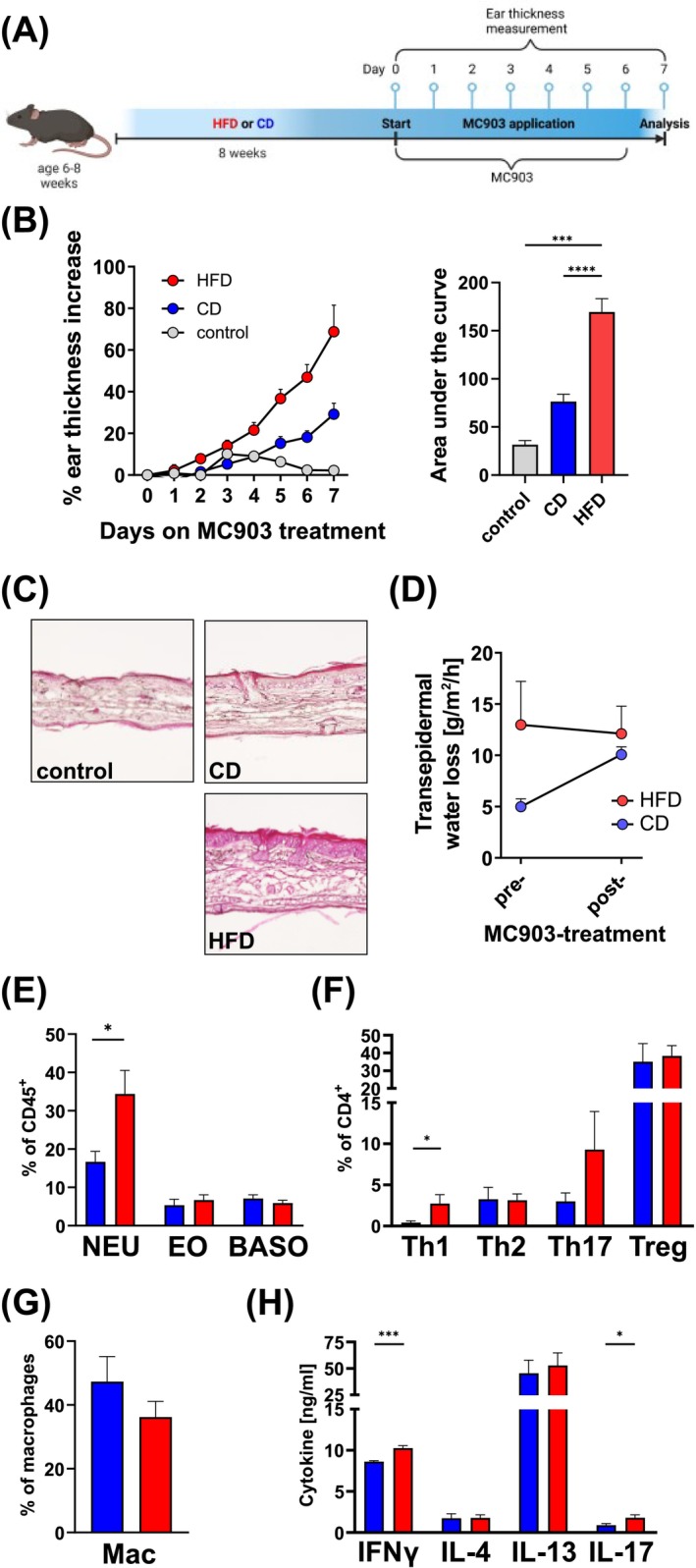
Obesity aggravates MC903‐induced dermatitis. (A) Schematic of mice on HFD or CD treated with topical MC903‐application. Created with BioRender.com. (B) Change (% of starting thickness) of ear thickness of untreated mice (gray) or mice treated with MC903 on HFD (red) or CD (blue). Bars show the area under the curve with 12 mice per group from three independent experiments. ***p < 0.001, ****p < 0.0001; one‐way ANOVA. (C) Representative photomicrographs of hematoxylin and eosin (H&E) stained ear skin sections of untreated (top left), and MC903‐treated mice on CD (top right) or HFD (bottom right). (D) TEWL measurement pre‐ and post‐MC903 treatment. Graph shows the mean + SD of 4 mice per group from one of two independent experiments. (E‐G) Flow cytometric analysis of neutrophils (NEU), eosinophils (EO) and basophils (BASO) (E), T helper cell subsets (F) and alternatively activated macrophages (AAM) (G) and in the ear skin of MC903‐trated (day 7) mice kept on CD (blue) or HFD (red). (H) IFNγ‐, IL‐4‐, IL‐13‐ and IL‐17A‐production of restimulated (anti‐CD3/anti‐CD28; 72 h) skin‐draining lymph nodes determined by ELISA. Bars show the mean + SEM of 8 mice per group from two independent experiments (E, H) or the mean + SD of 4 mice per group from one experiment (F). *p < 0.05, Student's *t*‐test.

It has been previously reported that mice on HFD developed a more Th17‐ and Th1‐driven immune response in a model of AD‐like inflammation [13]. Thus, we utilized the MC903‐induced model of AD‐like inflammation, which shares many characteristics with human AD such as eczema, redness, swelling, as well as recruitment of cell populations, to mice after 8 weeks on HFD or CD (Figure 4A). We applied MC903 (Vitamin D3 analogue; calcipotriol) in ethanol daily to the dorsal side of the ear pinnae and measured ear thickness over the course of 7 days. Strikingly, MC903‐treated obese animals had a 68.8% increase in ear thickness after treatment, which was highly significant (*p* < 0.0001), compared to lean control animals (29.2% increase) (Figure 4B,C). This indicated that the impaired skin barrier in obese mice leads to deeper penetration of MC903 relative to lean animals. Indeed, on day 7 of MC903 treatment, we observed an increase of infiltrating cells as well as a thickening of dermal and epidermal layers in obese animals, while the increase in lean animals was only moderate at this time point (Figure 4C). As shown above, while the obese mice had significantly elevated TEWL before application of MC903, reflecting the compromised skin barrier, however, after treatment both groups showed a similar TEWL indicating that the HFD‐induced skin barrier disruption is comparable to the breakdown in skin barrier of lean mice treated with an inflammatory compound (Figure 4D). When we identified cell populations in the inflamed skin, we observed an increased influx of neutrophils (16.7% on CD vs. 34.4% on HFD; *p* < 0.05) in obese animals (Figure 4E) as well as an aggravation of the pro‐inflammatory Th1 (0.05% on CD vs. 2.4% on HFD; *p* < 0.05) and Th17 (2.9% on CD vs. 9.3% on HFD) cells in the ear skin (Figure 4F). AAM were only non‐significantly reduced during HFD (Figure 4G). Analysis of cytokines supports the shift towards mixed Th1/2/17 response as reflected by increased levels of IFNγ and IL‐17 during HFD, while Th2‐associated cytokines IL‐4 and IL‐13 were comparable between mice on CD and HFD (Figure 4H).

### 3.5 Increased Skin IL‐17 Further Dysregulates Skin Barrier Proteins In Vitro

**FIGURE 5:**
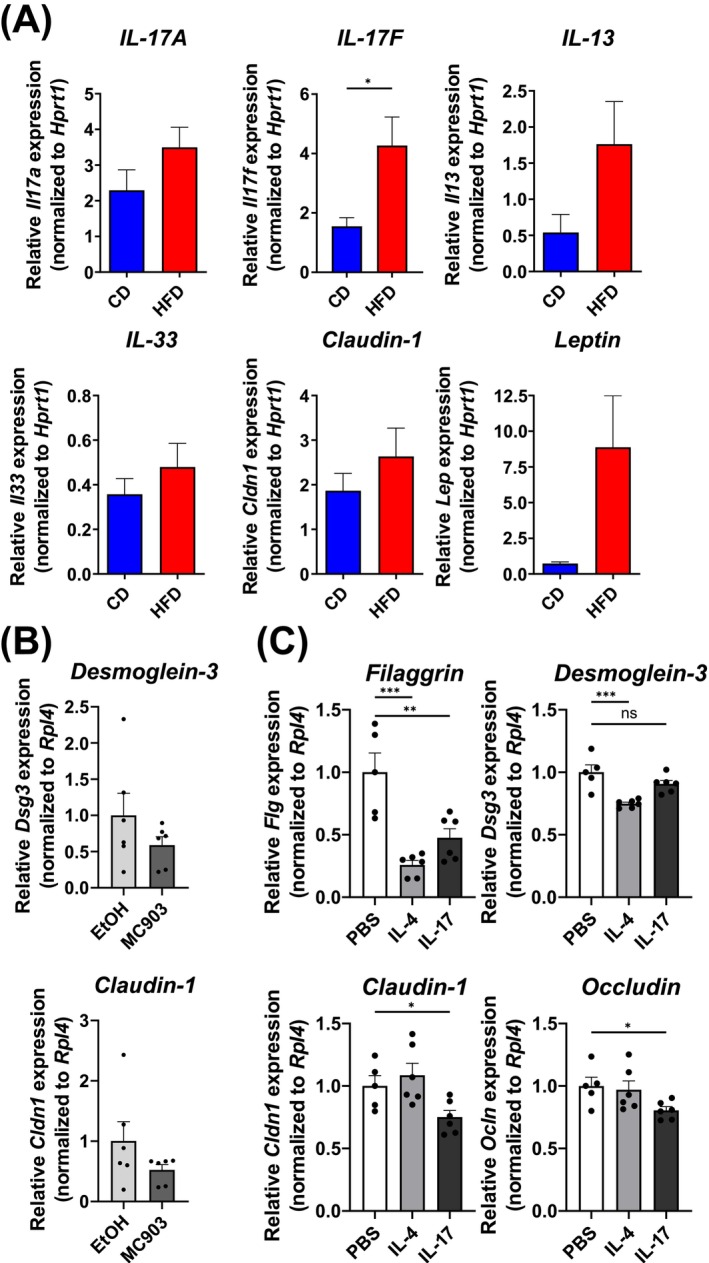
IL‐17 promotes downregulation of skin barrier proteins in keratinocytes. (A) Gene expression of indicated genes in the ear skin of mice on CD (blue) or HFD (red) treated with MC903. Bars show the mean + SEM of 8 mice per group from three independent experiments. *p < 0.05, Student's *t*‐test. (B) Skin explants were obtained from abdominal skin of naive wildtype mice and cultured in vitro. Skin explants were treated with ethanol (vehicle control, light gray) or MC903 (4 nmol, dark gray) daily and transcripts of *desmoglein‐3* and *claudin‐1* were analyzed after 3 days. (C) Keratinocytes were cultured in vitro and stimulated with PBS (vehicle control, open bars), IL‐4 (light gray) or IL‐17A (dark gray) for 24 h. Transcripts of indicated genes were analyzed by qPCR. Bars show the mean + SEM of 5–6 biological replicates per group from two independent experiments. *p < 0.05, **p < 0.01, ***p < 0.001; one‐way ANOVA.

We and others have previously highlighted the role of IL‐17 and its receptor as an important cytokine in skin inflammation during AD and the atopic march [25, 26]. While we did not observe major changes in the T cell‐derived IL‐17 in skin‐draining lymph nodes under steady‐state conditions, we could observe increased transcripts of *Il17a* and *Il17f* alongside *Il13* and *Leptin* in the skin of obese mice that had been treated with MC903 (Figure 5A), while *Il33* transcripts were comparable. Interestingly, we observed upregulation of *Claudin‐1* transcripts, indicating an attempt to restore barrier function under inflammatory conditions (Figure 5A). Skin explants treated in vitro for 3 days with MC903 showed that MC903 on its own can decrease skin barrier function (Figure 5B), but did not lead to increased expression of IL‐17 (data not shown). Thus, the application of MC903 may lead to the breakdown of important skin barrier proteins, which is even more pronounced in obese mice with an IL‐17‐biased environment. The observed increase of *Il17a* and *Il17f* expression led us to speculate that the presence of IL‐17 in the skin could modulate skin barrier function by acting directly on keratinocytes. Therefore, we analyzed keratinocytes derived from adult tail skin. We could confirm that IL‐17 treatment of keratinocytes causes the downregulation of skin barrier protein transcripts including *filaggrin*, *occludin*, and *claudin‐1* (Figure 5C). Thus, we conclude that in the type 17‐biased skin environment of obese mice, there is an increased production of IL‐17A in response to inflammatory stimuli, which further aggravates the breakdown of the skin barrier by directly acting on keratinocytes.

### 3.6 Increased Allergic Sensitization in Mice on High‐Fat Diet Promotes Food Allergy

**FIGURE 6:**
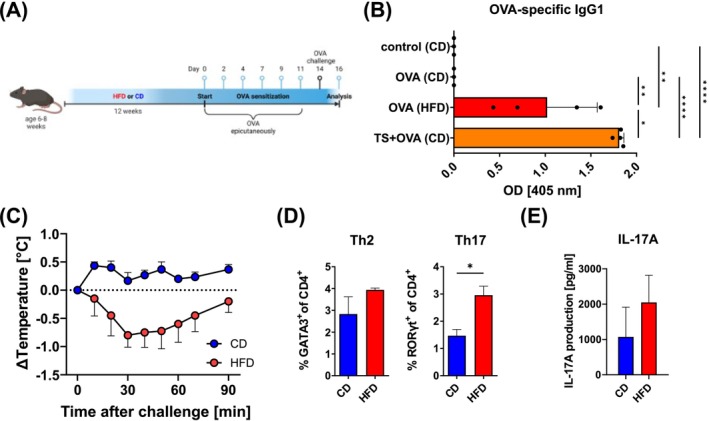
Obesity facilitates allergic sensitization in mice. (A) Schematic of mice on HFD or CD treated with epicutaneous ovalbumin‐application. Created with BioRender.com. (B) ELISA of OVA‐specific IgG1‐antibodies in the serum of control mice (untreated, CD), mice on CD (blue bars) or HFD (red) treated with ovalbumin epicutaneously and mice on CD that were tape‐stripped before OVA application (TS, orange bars). Bars show the mean + SD from one out of two representative experiments with 3–4 mice per group. *p < 0.05, **p < 0.01, ****p < 0.0001; one‐way ANOVA. (C) Mice on CD (blue) or HFD (red) topically treated with OVA were orally challenged with OVA and temperature was recorded on indicated time points and calculated as change to the body temperature before challenge. *, *p* < 0.05, Student's *t*‐test of area under the curve. (D) Frequency of Th2 and Th17 subsets in the auricular lymph nodes of mice on CD (blue) or HFD (red) treated with OVA epicutaneously. (E) IL‐17‐ELISA of restimulated auricular lymph nodes from (D). (C, D) Bars show the mean + SD of 3–4 mice per group from one out of two representative experiments. *p < 0.05, Student's *t*‐test.

To determine if the impaired skin barrier of obese mice leads to cutaneous and systemic allergic sensitization, we applied the model antigen ovalbumin (OVA)—without additional adjuvants—to the ear skin of mice on CD or HFD (Figure 6A). The increased permeability of the skin of obese mice was sufficient for topical application of OVA to elicit an OVA‐specific immune response, as we were able to detect OVA‐specific IgG1 in obese animals but not in lean control mice with an intact skin barrier (Figure 6B). Indeed, the levels of OVA‐specific IgG1 detected in obese mice were comparable to lean mice which had their skin barrier disrupted by mechanical damage via tape‐stripping (Figure 6B). To further test if the elevated antibody response to OVA in obese mice treated with allergen on the skin led to functional systemic sensitization, we challenged mice with a single oral dose of OVA and measured changes in body temperature (Figure 6A,C). While the body temperature of mice on CD remained constant, we observed a significant drop in temperature of mice on HFD, indicating signs of anaphylaxis due to systemic OVA‐specific sensitization (Figure 6C). In addition, in the skin‐draining lymph nodes of mice on HFD, there were elevated T cell responses, including both Th2 and Th17 cells (Figure 6D). Consistent with a Th17 environment, there was increased IL‐17 production in OVA‐challenged obese mice (Figure 6E).

Taken together, our results indicate that obesity impairs the skin barrier, which leads to a type 3‐biased immune environment in the skin that, upon allergic sensitization, leads to AD‐like inflammation and subsequently food allergy.

## 4 Discussion

The relationship between obesity and skin barrier dysfunction has garnered significant attention in recent years, with obesity being associated with various skin inflammatory conditions such as psoriasis and atopic dermatitis, where the skin barrier is often disrupted and poses a risk factor for allergy development.

Previous studies observed that persons living with obesity (PWO) have a significantly higher TEWL than persons without obesity [27]. In a recent study, authors demonstrated a strong association of the body mass index with TEWL and skin pH and suggested a causal relationship of obesity and TEWL [28], which may extend to inflammatory skin conditions beyond AD (reviewed in [29]). In mice, studies found that diet‐induced obesity can impair keratinocyte expression of skin barrier proteins, which are critical for maintaining skin integrity and preventing allergic sensitization [30]. We now show that obesity induces a dysregulated skin barrier that increases the risk of allergic sensitization through the skin. The compromised skin barrier observed in our obese mice likely leads to enhanced allergen penetration and thus elicits a stronger inflammatory response. This is particularly relevant as obese mice show altered expression of proteins associated with skin integrity and functions, including claudin‐1, filaggrin, desmoglein‐3, and involucrin.

Dysfunction in claudin expression has been linked to increased permeability of the skin barrier, allowing allergens to penetrate more easily and leading to allergic sensitization [31]. In the small intestine, HFD causes restructuring of the tight junctions with downregulation of claudin‐1, while claudin‐2 was upregulated—suggesting that claudin‐1 downregulation is part of a broader mechanism of claudin switching in response to dietary fat intake [32]. As this mechanism directly contributes to obesity‐associated increased intestinal permeability, this may also affect skin permeability. Indeed, claudin‐1 was previously shown to contribute to skin barrier integrity, and alterations of claudin‐1 lead to increased TEWL [33]. Claudin‐1 deficiency in mice leads to severe dehydration and compromised skin barrier function [34], with claudin‐1 downregulation being a significant factor in the pathogenesis of atopic dermatitis [35]. This is particularly relevant in the context of obesity, where systemic inflammation may exacerbate skin barrier dysfunction. Gutowska‐Owsiak et al. highlighted the role of inflammatory cytokines, such as IL‐17, in downregulating claudin‐1 expression in keratinocytes, aggravating the already compromised skin barrier even more [36], which we could observe in keratinocyte cultures. The correlation between claudin‐1 expression and skin barrier integrity underscores the importance of maintaining tight junction protein levels for optimal skin health [36]. Similarly, the breakdown of desmosomes compromises the structural integrity of the skin [37], facilitating allergen entry, as demonstrated in studies where impaired skin barrier function was associated with increased allergen sensitization and subsequent allergic reactions [16, 38]. *Desmoglein‐3* plays an important role in maintaining keratinocyte cohesion and signaling pathways that regulate cell adhesion, thereby influencing keratinocyte behavior, including migration and wound healing [39]. Interestingly, *Desmoglein‐1* appears not to be affected by obesity. Whether different transcriptional regulatory networks actively maintain *Dsg1* expression, thereby preventing complete desquamation or epidermal breakdown, will be the subject of further studies.

Filaggrin mutations have been extensively studied in relation to skin barrier dysfunction and allergic diseases. Loss‐of‐function mutations in the filaggrin gene result in disrupted skin barrier function, predisposing individuals to eczema and enhancing the risk of allergic sensitization and maintenance of food allergy [40, 41]. Previous results from our lab showed that both *Filaggrin* and *Tmem79* mutations can contribute to AD development as well as atopic march [26, 42]. The presence of allergens in the skin can trigger immune responses that lead to systemic allergic reactions, as evidenced by increased levels of immunoglobulin E (IgE) and inflammatory cytokines in sensitized individuals [43, 44].

Obesity is characterized by a state of chronic low‐grade inflammation, and while it does not per se cause overt skin inflammation, it predisposes one to a type 3‐prone immune response, which, upon allergic sensitization, leads to a mixed Th1/2/17 response. Supporting our findings, it has been previously reported that mice on HFD developed a more Th17‐ and Th1‐driven immune response in the MC903 model of AD‐like inflammation [13]. In the study by Bapat et al., the authors showed that HFD shifted the classical skin Th2 response to MC903 application towards a Th17‐biased proinflammatory response. Importantly, however, our temporal analysis reveals that skin barrier defects precede the type 3‐biased immune status, as downregulation of skin barrier proteins was observed as early as 2 weeks after HFD initiation, while elevated IL‐17 levels occurred only during MC903 treatment, and T cells did not produce IL‐17 upon restimulation under steady‐state conditions. We and others have previously highlighted the role of IL‐17 and its receptor as an important cytokine in skin inflammation during AD and the atopic march [26, 42]. Our data now extend these observations by showing that in the type 3‐biased skin environment of obese mice, there is an increased production of IL‐17A and IFNγ alongside the classical type 2 response against inflammatory stimuli, which further aggravates the breakdown of the skin barrier by directly acting on keratinocytes. These cytokines have been shown to downregulate the expression of key barrier proteins, including filaggrin and claudin‐1, thereby exacerbating skin barrier dysfunction [16, 30]. Indeed, our data support that IL‐17 can downregulate claudin‐1, desmoglein‐3, and filaggrin in keratinocytes and skin explant cultures in vitro, as well as in HFD‐fed mice.

Importantly, as we and others observed, AD following the breakdown of the skin barrier can lead to systemic effects, such as airway inflammation or food allergy [26, 45], usually described as atopic march. We have shown that IL‐17A is a critical effector cytokine that promotes airway inflammation, probably secreted by γδ T cells [26]. In the present study, the source of IL‐17 in the skin remains elusive; however, we speculate that Th17 cells [13], γδ T cells [26], as well as ILC3 [46] contribute to the observed obesity‐induced inflammatory shift. Previous data have already outlined the mechanism of epicutaneous sensitization leading to food allergy via the involvement of IL‐4‐responsive skin dendritic cells, basophils, ILC2, and mast cells [47, 48, 49, 50]. Importantly, the breakdown of the skin barrier may also contribute to other obesity‐associated comorbidities, such as impaired wound healing and bacterial infections [51].

Aligning with our data, obesity impacts the microbiomes of both the gut and skin. In the gut, obesity is associated with reduced microbial diversity and shifts in the balance of bacterial phyla, such as an increase in Firmicutes and a decrease in Bacteroidetes. Antibiotic exposure, particularly at subtherapeutic levels, exacerbates these effects, promoting weight gain and metabolic dysregulation by altering gut microbial composition [52, 53, 54]. On the skin, obesity alters microbial diversity and promotes colonization by specific taxa like *Corynebacterium* and *Finegoldia*, particularly in areas with increased skin folds and moisture. These changes are linked to heightened inflammation, impaired wound healing, and susceptibility to infections [51, 55]. In our hands, antibiotic treatment of mice on HFD did not prevent the weakening of the skin barrier.

While our study provides novel insights into the pathogenesis of allergy, it has certain limitations. Foremost, the study was conducted in mice, which, for example, have a thinner epidermal layer and a higher density of hair follicles. Yet, based on human studies that show increased TEWL in PWO, we will pursue our findings using clinical samples in future studies. Secondly, the mechanism through which diet and/or obesity modulate skin barrier protein expression is still unknown and subject to current research. Here, adipokines may play an important role, which may be critical mediators linking obesity to AD (reviewed in [56]). Dysregulation of adipokines, such as elevated leptin and decreased adiponectin, is observed in PWO and patients with AD. Notably, leptin promotes Th1 and Th17 cell differentiation while suppressing regulatory T cell proliferation, potentially explaining the Th17‐biased immune response we observed in our obese animals. Furthermore, the finding that obesity may shift AD towards a more Th17‐dominant phenotype, as suggested by studies showing 6.5‐ and 11.5‐fold increases in IL‐17A and IL‐17F‐positive T cells, respectively, in obese mice [13], aligns with our observed Th17‐biased immune environment.

The interplay of obesity and skin barrier function is an area of growing interest, particularly given the rising prevalence of obesity and its systemic impact on health. Obesity may compromise skin barrier integrity in PWO through several potential mechanisms. Firstly, the physical expansion of adipose tissue can lead to mechanical stretching of the skin. Such stretching may disrupt the structural organization of keratinocytes, impairing their ability to maintain an intact barrier [28], increasing TEWL, which has been reported to be significantly higher in obese individuals compared with their normal‐weight counterparts [4, 57]. Moreover, the inflammatory milieu associated with obesity plays a critical role in skin barrier impairment. Adipose tissue in obese individuals secretes a variety of pro‐inflammatory cytokines, including TNF‐α and IL‐6, which can exacerbate inflammation within the skin [30]. The resulting inflammation can lead to dermal edema and further compromise barrier function, creating a cycle of dysfunction. Additionally, obesity increases oxidative stress levels, which can contribute to skin barrier impairment. Elevated levels of reactive oxygen species (ROS) have been shown to alter cellular signaling in keratinocytes, which may affect skin hydration and barrier function [30].

The clinical implications of these findings are profound, as PWO with a compromised skin barrier may experience more severe allergic reactions and skin conditions, necessitating a comprehensive approach to treatment that includes both dermatological and metabolic considerations. Therapeutically, enhancing the expression of claudins and filaggrin through topical treatments or dietary interventions could help restore skin barrier integrity in obese patients. Probiotics and other dietary supplements have also been explored for their potential to improve skin barrier function and reduce inflammation, indicating a promising area for future research and clinical application.

In summary, we propose a sequential model where obesity‐induced downregulation of skin barrier proteins creates a compromised barrier that subsequently facilitates the shift towards an IL‐17‐prone skin microenvironment, which then aggravates allergic inflammation and further weakens skin integrity, creating a feed‐forward cycle that facilitates both cutaneous sensitization and the development of systemic food allergy.

## Author Contributions

A.M. designed, performed and analyzed experiments. A.D., L.S. and C.V. contributed to specific experiments. R.G.G. and J.P. performed 16S sequencing and bioinformatic analysis. P.G.F. provided essential reagents and animals, and edited the manuscript. C.S. conceptualized the study; designed, performed and supervised experiments; and wrote and revised the manuscript.

## Conflicts of Interest

The authors declare no conflicts of interest.

## Supporting information


Data S1.

## Data Availability

The data that support the findings of this study are openly available in NCBI at http://www.ncbi.nlm.nih.gov/bioproject/1299357, reference number PRJNA1299357.
