# Supplementary material for: Obesity Impairs Skin Barrier Function and Facilitates Allergic Sensitization in Mice
Source: Allergy. 2025 Sep 23;81(2):498–512. doi: 10.1111/all.70067 (PMC12862518; doi:10.1111/all.70067)
Supplement: Supplementary file 1 — Data S1. [file ALL-81-498-s001.pdf]

Supplementary Figure 1

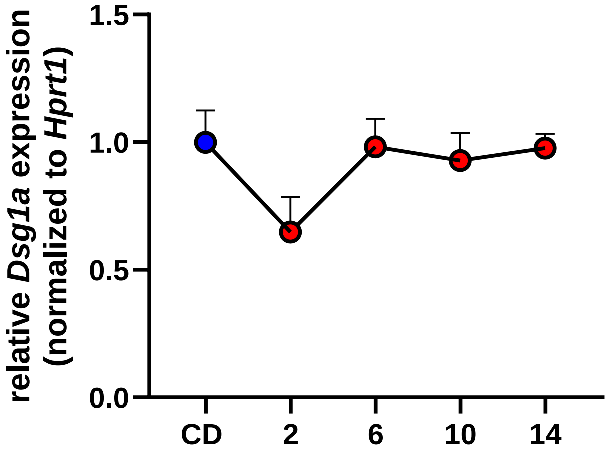

Expression of *Desmoglein-1* (*Dsg1*) in the ear skin of mice kept on CD (blue) or HFD (red) at the indicated time points. Depicted is the mean+SEM of 6-12 mice per group from two independent experiments.

Supplementary Figure 2

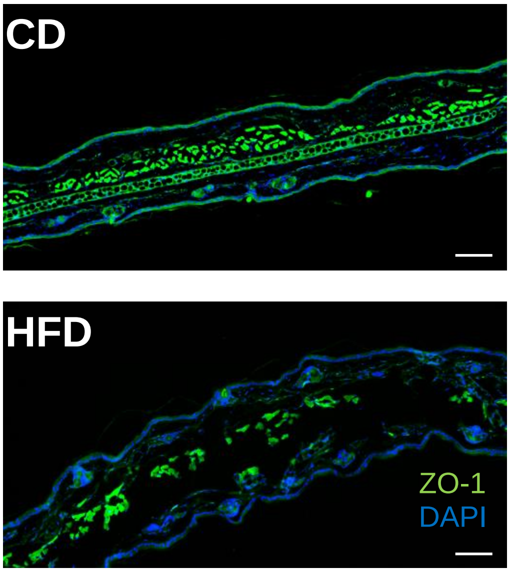

Immunofluorescence staining of cryo-sections of ears from mice kept on CD or HFD. Green=ZO-1, blue=DAPI. Scale bar = 100 µm.

Supplementary Figure 3

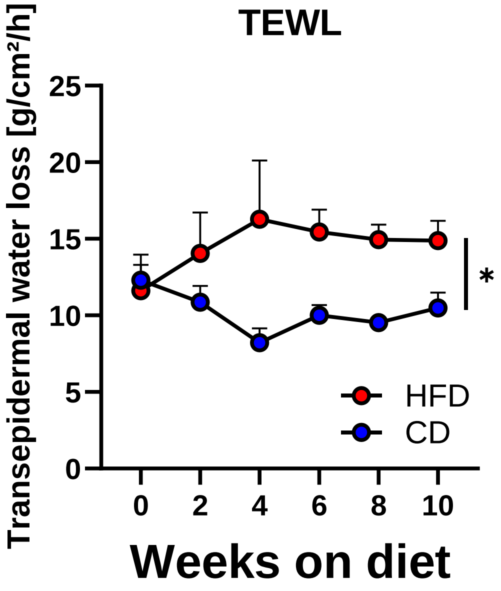

Transepidermal water loss (TEWL) measurement of mice kept on CD (blue) or HFD (red) for the indicated duration. Bars show the mean+SEM of 6 mice per group from two independent experiments. *, $p<0.05$; Student t-test of area under the curve.
